# Detection of Atypical Porcine Pestivirus in Piglets from Danish Sow Herds

**DOI:** 10.3390/v13050717

**Published:** 2021-04-21

**Authors:** Kasper Pedersen, Charlotte Sonne Kristensen, Bertel Strandbygaard, Anette Bøtner, Thomas Bruun Rasmussen

**Affiliations:** 1 SEGES Danish Pig Research Centre, Agro Food Park 15V, DK-8200 Aarhus N, Denmark; CSK@seges.dk; 2 Department of Virus & Microbiological Special Diagnostics, Statens Serum Institut, Artillerivej 5, DK-2300 Copenhagen S, Denmark; BSTR@ssi.dk (B.S.); ANEB@sund.ku.dk (A.B.); TBRU@ssi.dk (T.B.R.); 3 Department of Veterinary and Animal Sciences, Faculty of Health and Medical Sciences, University of Copenhagen, DK-1870 Frederiksberg C, Denmark

**Keywords:** epidemiology, atypical porcine pestivirus (APPV), congenital tremor (CT), piglets

## Abstract

Atypical porcine pestivirus (APPV) was first discovered in North America in 2015 and was later shown to be associated with congenital tremor (CT) in piglets. CT is an occasional challenge in some Danish sow herds. Therefore, we initiated an observational case control study to clarify a possible relationship between CT and APPV in Danish pig production. Blood samples were collected from piglets affected by CT (*n* = 55) in ten different sow herds and from healthy piglets in five sow herds without a history of CT piglets (*n* = 25), as well as one sow herd with a sporadic occurrence of CT (*n* = 5). APPV was detected by RT-qPCR in all samples from piglets affected by CT and in three out of five samples from piglets in the herd with a sporadic occurrence of CT. In the herds without a history of CT, only one out of 25 piglets were positive for APPV. In addition, farmers or veterinarians in CT-affected herds were asked about their experience of the issue. CT is most often seen in gilt litters, and a substantial increase in pre-weaning mortality is only observed in severe cases. According to our investigations, APPV is a common finding in piglets suffering from CT in Denmark.

## 1. Introduction

Atypical porcine pestivirus (APPV; species *Pestivirus K*) was first discovered in North America in 2015 using metagenomics [1], but it has been retrospectively detected in serum collected from fattening pigs in Switzerland in 1986 [2]. Due to this relatively new discovery, and since this virus does not seem to cause losses at the same level as, e.g., porcine reproductive and respiratory syndrome virus and porcine circovirus, little is known about its epidemiological properties. Recent publications show that the virus has been detected in several other countries, including Austria [3], Brazil [4], Canada [5], China [6], Denmark [7], Germany [8,9], Hungary [10], Italy [11], Korea [12], the Netherlands [13], Spain [14], Sweden [15,16], Switzerland [2], Serbia [9], Taiwan [9], the United Kingdom [9], and the United States [1,17]. The virus has a high genetic variability between countries as well as within countries [4,9,18,19], and recombination between APPV strains has recently been reported in China [18]. APPV belongs to the genus *Pestivirus*, within the family *Flaviviridae* [20]. Members of this family share morphological properties in terms of positive-sense single-stranded RNA genomes, envelope formation, and they all have the same open reading frame strategy in genome replication [1,21,22]. 

In 2016, APPV was associated with congenital tremor (CT) in piglets [13,17,23]. CT-affected piglets have been shown to have a varying degree of hypomyelination in the brain and spinal cord [3,5,23,24]. These tremors, which are defined as an involuntary quivering have been documented to varying degrees in piglets with CT. In severe cases, the disease can cause difficulty in suckling. Malnutrition, insufficient maternal immunity, and crushing by the sow because of starvation may explain the underlying reason for the elevated pre-weaning mortality sometimes seen in litters affected by CT [3,5,13,25]. In litters comprising both apparently healthy and CT-affected piglets, the mortality rate has been shown to be 24.6%, 17.2%, and 26.0% (*n* = 5, 41 and 48) compared to 12.7%, 6.5%, and 11.0% (*n* = 15, 50 and 183) in healthy litters, respectively [5,13,25]. In CT-affected piglets alone, the mortality rate has been as high as 46.4% [25].

Since CT is an occasional challenge in Danish sow herds, we initiated an observational case control study to clarify a possible relationship between CT and APPV infection in Danish pig production. We also asked the farmers or veterinarians included in the study about their experiences with CT in their herds.

## 2. Materials and Methods

The study was performed as an observational case control study in 16 Danish sow herds geographically distributed throughout Denmark (Table 1). Case herds (*n* = 10) were defined as herds with an acute onset of CT in at least five different litters. Control herds were selected based on not having piglets with symptoms of CT for a period of at least one year. Furthermore, an intermediate herd with an occasional occurrence of CT was included. The study was performed in the period from June 2019 to April 2020. Since CT is an occasional occurrence, we requested herds with CT-affected piglets on our website (www.svineproduktion.dk, 18 July 2019), and farmers or veterinarians were encouraged to contact us if they had piglets with CT in their herd. The veterinarians responsible for each case herd were asked if they were aware of possible control herds. 

In each case herd, blood samples were collected from five CT-affected piglets in five different litters in the farrowing unit. In both the control herds and the intermediate herd, blood samples were taken from 1 healthy pig per litter in 5 different gilt litters. The intermediate herd was initially included as a control herd, but during the visit to the herd, low degree CT was observed in a few piglets. However, these were not sampled due to low degree insignificant CT. The blood samples were taken from the Vena jugularis into BD Vacutainer^®^ serum tubes with coagulation activator Hemogard™. The blood samples were stored at 5.0 °C until they were transported to the Department of Virus & Microbiological Special Diagnostics at Statens Serum Institute within 24 h of sampling. 

Serum was separated from the full blood by centrifugation at 3500 rpm for 5 min. All serum samples were analyzed individually and in pools by RT-qPCR. Viral RNA was purified using a MagNA Pure 96 robot and the DNA and Viral NA Small Volume kit (Roche Diagnostics, Hvidovre, Denmark). Two RT-qPCRs targeting the coding sequence for the nonstructural protein NS5B were used for the studies. First, a broad range pestivirus-specific RT-qPCR using the primers Pesti-11453-F (5′-ACA GCM ATR CCA AAR AAT GAG AA-3′) and Pesti-11607-R (5′-TTT CTG CTT TAC CCA VTT RTA CAT-3′) from Beer et al. 2017 [8], together with the Qiagen OneStep RT-PCR kit (Qiagen, Hilden, Germany) and Resolight dye (Roche Diagnostics), was used to determine the level of viral RNA expressed as cycle threshold (Cq)-values. Second, positive samples were confirmed by an APPV-specific RT-qPCR targeting the NS5B using the primers APPV-NS5B-303F (5′-GTA GGG CGG ATA CAG AAA TA-3′) and APPV-NS5B-385R (5′-GGY ACT TCC TCC ATC ATG G-3′) and the probe APPV-NS5B-336-FAM (5′-FAM-AAA TAT TGG AAA TYY ATT GAC AAT TTG AC-BHQ1-3′) as described [8].

Furthermore, the farmers or veterinarians in the case herds were asked to register any symptoms of tremor, for how long CT had been present in the herd, parity of the sows having CT-affected litters, and an assessment of the mortality rate among CT-affected piglets and the strategy used for acquiring new breeding stock to the herd. In the control herds, the farmers were asked about their most recent experience with CT in piglets and about their strategy for acquiring new breeding stock.

Results were collected in Microsoft® Excel® Office 365 MSO (16.0.12527.20612) 32-bit, and tables were also made in this program. The results from RT-qPCR were processed in GraphPad Prism Version 9.1.0 and presented in figure. 

## 3. Results

### 3.1. Detection of APPV

The study included a sample set of serum from 55 CT-affected piglets collected in ten case herds, 25 healthy piglets from five herds without a history of CT-affected piglets, and five piglets without CT from a herd categorized as an intermediate herd (Table 1 and Table S1). All piglets came from different litters in the farrowing unit and ranged in age from 2 to 31 days.

**Figure 1:**
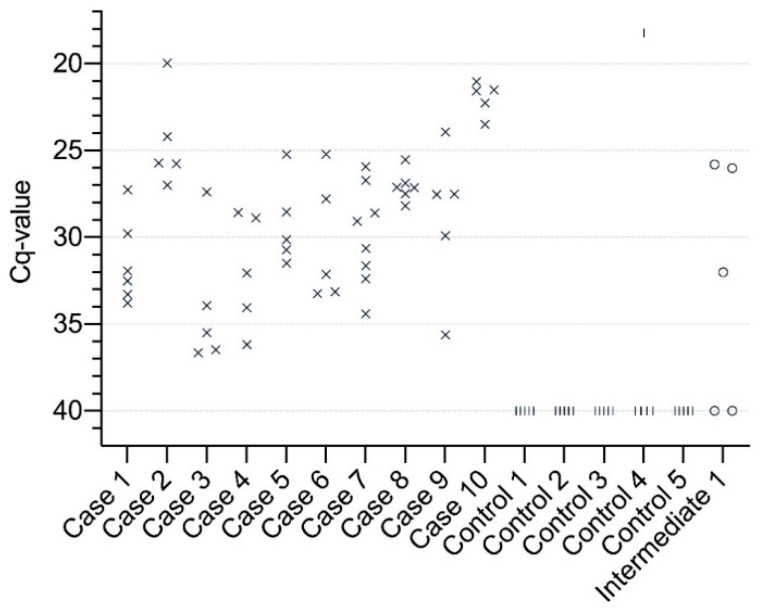
Results from the qRT-PCR analysis given as the Cq-value for each sample. Each sample in the ten case herds is indicated as “×”, in the control herds as “l”, and in the intermediate herd as “o”. Negative values are given as Cq = 40.0, which is the limit value in test. Individual Cq-values are presented in Table S1.

APPV was detected in all 55 piglets (100%) suffering from CT in the case herds with Cq-values ranging from 20.0–36.7 (Table 1, Figure 1 and Table S1). In the five control herds without incidence of CT, one single piglet out of the 25 (4%) healthy piglets was positive for APPV. Apparently, this piglet had the lowest Cq-value at 18.2 (Figure 1 and Table S1). In the intermediate herd, three out of five piglets (60%) that did not suffer from CT were positive for APPV, with Cq-values ranging from 25.8 to 30.2 (Table 1, Figure 1 and Table S1). 

### 3.2. Survey

Answers to the questions about CT presentation in each herd are listed in Table 2.

CT was described with a different pattern among the case herds. Two farmers described the tremor to stop when the piglets were asleep and how they most often showed symptoms of tremor during stressful periods such as when being vaccinated or examined. Another farmer described how the piglets with tremor sometimes appeared tired, probably as a result of the tremor itself, and how the sow could accidentally crush them. In three case herds, the piglets’ tremor was so pronounced that they jumped up on their front or hind limbs. It was hypothesized by the farmers or veterinarians that piglets with these severe symptoms would probably have difficulty suckling. From a veterinarian point of view, this entails a risk of impaired uptake of protective antibodies and not least a decreased nutrient uptake. However, only two of the farmers or veterinarians from the case herds experienced increased mortality among the CT-affected piglets. Most often, the piglets stopped shaking before weaning, but one farmer had a few 30 kg pigs rejected for export due to severe tremor in accordance with livestock transportation rules.

## 4. Discussion 

The finding of APPV in serum from CT-affected piglets is consistent with other studies [1,2,3,6,7,13,14,17,23,25,26]. In the control herds without a history of CT-affected piglets, we detected APPV in one piglet (4%), which, compared to the prevalence of APPV in piglets from the case herds (100%), suggests that APPV is involved in the pathogenesis of developing clinical symptoms of CT in piglets. This is consistent with results in other studies [1,13,17,23]. One other study investigated serum samples (*n* = 7) from a herd without a history of CT, and found all samples APPV-negative [13]. However, the finding of APPV in one piglet from the control herd in this study indicates that APPV can circulate in herds without causing clinical disease with CT. Interestingly, this piglet had the lowest Cq-value and thus the highest viral load of APPV in serum (Figure 1 and Table 1). The piglet was 11 days old, originating from a gilt in a 950-sows herd, they were producing their own breeding stock and have not experienced CT for 15–20 years. However, there seems to be contradictory results in this context. De Groof et al. (2016) did not find any relationship between the virus concentration in serum and the severity of CT [13], whereas the statistical results of Sutton et al. (2019) suggested a possible association between APPV viral titre and the presence of CT [25]. APPV has, as mentioned above, commonly been detected in serum from healthy piglets in case herds in other studies [8,9,13,23]. This study included a type of intermediate herd, in which we detected APPV in three apparently healthy piglets, along with the presentation of low degree CT-affected piglets in the farrowing unit, and the farmer did not experience any CT until sample collection. A reasonable explanation for the detection of APPV in piglets without CT is that they are horizontally infected without developing clinical signs of CT. Unfortunately, no blood samples were collected from the pigs with CT in the intermediate herd. Horizontal infection with APPV was seen in one study in connection with co-mingling of infected and uninfected pigs on the flat deck after weaning [26]. Otherwise, regarding the development of CT in piglets, the influence of the time of the infection during pregnancy of the gilt/sow is an interesting topic to investigate in the future.

In this study, CT was most often observed in piglets from gilts (Table 2). Therefore, blood samples were collected from piglets standing by gilts, except in one herd that also experienced CT piglets from older sows. This observation is in line with observations from other studies [4,13], although a recent study has shown a case of a higher incidence of CT in litters from second and third parity sows compared to first and fourth parity sows [18]. A higher presentation in gilt litters suggests immunity in breeding animals after infection. Cagatay et al. (2019) have demonstrated how horizontal infection of APPV resulted in the development of protective immunity against APPV, mainly based on high neutralizing E2-specific antibodies [26]. It was concluded that this can be beneficial in establishing herd immunity. However, the duration of immunity to APPV needs to be investigated further in future studies.

Several studies argue that APPV can be recurrent because of persistently infected animals in the herd [3,13,14,23]. Even though persistently infected animals have a reduced viral load of APPV in serum, they still seem to shed virus in feces, serum, and oral fluids [13]. Half of the herds included in this study introduce breeding animals from other herds. This often occurs around the time of breeding. APPV-naive animals may then be introduced to APPV in the new herd and might therefore be viremic with APPV during the gestation period. This entails the risk of foetuses being exposed to the virus through trans-placental transmission [13,17] and thus being born with CT. 

All statements in the survey study are based on subjective evaluation by the farmers and veterinarians. This may give rise to an erroneous assessment (misinformation bias) of the impact of CT and its presentation in each herd. Therefore, pre-weaning mortality caused by CT could be higher than that reported in this study, as published in other studies. Clearly, the participants in the case herds were aware that they had been included in the study as case herds, which is contrary to the general guidelines for observational case control studies. 

## 5. Conclusions

According to our investigations, APPV is a common finding in piglets suffering from CT in Denmark. Interestingly, APPV was detected in one healthy piglet from a herd without a history of CT. Since CT mainly occurs in gilt litters, immunization of the breeding animals should be addressed in future.

## Figures and Tables

**Table 1:** Summary of laboratory analyses of serum samples tested for APPV by RT-qPCR.

Sample Origin	Serum Positive/Tested (%)	Pool Positive/Tested (%)	Date of Sample Collection	Region
Case 1	6/6 (100)	1/1 (100)	7 August 2019	Southern Denmark
Case 2	5/5 (100)	1/1 (100)	21 August 2019	Southern Denmark
Case 3	5/5 (100)	1/1 (100)	26 August 2019	Central Jutland
Case 4	5/5 (100)	1/1 (100)	5 September 2019	Northern Jutland
Case 5	5/5 (100)	1/1 (100)	14 October 2019	Northern Jutland
Case 6	5/5 (100)	1/1 (100)	14 November 2019	Southern Denmark
Case 7	8/8 (100)	1/1 (100)	5 December 2019	Zealand
Case 8	6/6 (100)	1/1 (100)	16 December 2019	Central Jutland
Case 9	5/5 (100)	1/1 (100)	23 December 2019	Northern Jutland
Case 10	5/5 (100)	1/1 (100)	10 January 2020	Southern Denmark
Control 1	0/5 (0)	0/1 (0)	27 January 2020	Central Jutland
Control 2	0/5 (0)	0/1 (0)	28 January 2020	Central Jutland
Control 3	0/5 (0)	0/1 (0)	28 January 2020	Central Jutland
Control 4	1/5 (20)	1/1 (100)	18 March 2020	Northern Jutland
Control 5	0/5 (0)	0/1 (0)	23 March 2020	Zealand
Intermediate	3/5 (60)	1/1 (100)	27 February 2020	Northern Jutland

**Table 2:** Overview of answers to questionnaires.

**Duration of CT Presentation in the Herd**	**Number of Herds**		
-Few sow batches	2		
-Every batch for a period of one to three months	6		
-Ongoing issue for six months	2		
**Parity of Sows with CT-Affected Litters**	**Number of Herds**		
-Gilts	9		
-1st–3rd parity	1		
-All parities	0		
**Strategy for New Breeding Stock**	**Case Herds**	**Control Herds**	**Intermediate Herd**
-Own breeding stock	2	2	-
-Own breeding stock, but supplied by other herds	3	-	-
-Purchased breeding animals from other herds	5	3	1

## Data Availability

The data presented in this study are available in https://doi.org/10.3390/v13050717 and Table S1.

## Supplementary Materials

The following are available online at https://www.mdpi.com/article/10.3390/v13050717/s1, Table S1: Results of laboratory analyses of serum samples tested for APPV by RT-qPCR given as the Cq-value for each sample.
